# Supplementary material for: Identifying Potent Nonsense-Mediated mRNA Decay Inhibitors with a Novel Screening System
Source: Biomedicines. 2023 Oct 16;11(10):2801. doi: 10.3390/biomedicines11102801 (PMC10604367; doi:10.3390/biomedicines11102801)
Supplement: Supplementary file 1 [file biomedicines-11-02801-s001.zip › biomedicines-2632802-supplementary.pdf]

	structures of 1a-k
**1a**	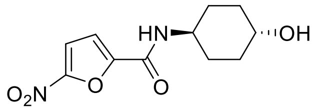
**1b**	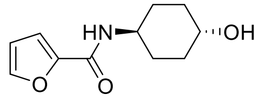
**1c**	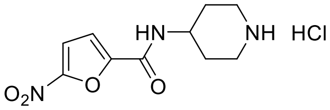
**1d**	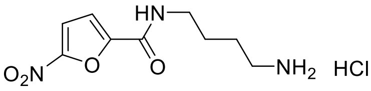
**1e**	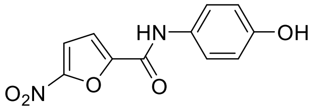
**1f**	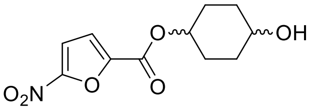
**1g**	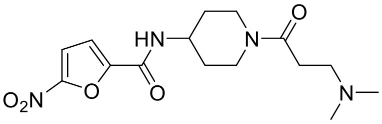
**1h**	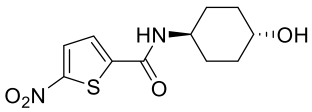
**1i**	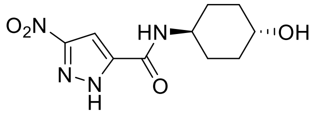
**1j**	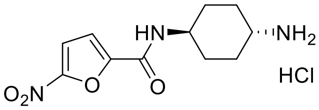
**1k**	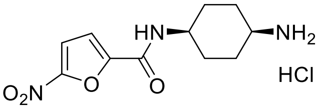

	structures of 2a-i
**2a**	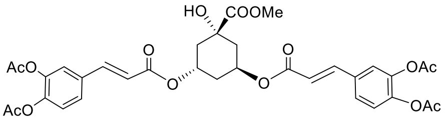
**2b**	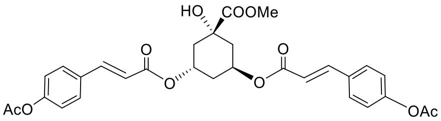
**2c**	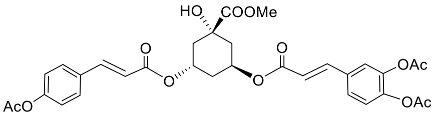
**2d**	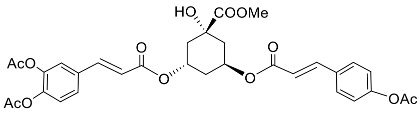
**2e**	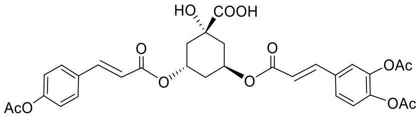
**2f**	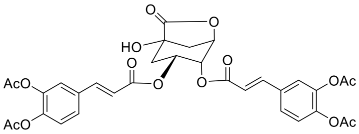
**2g**	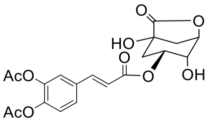
**2h**	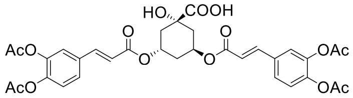
**2i**	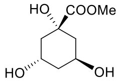

Supplemental Table S1
